# Ototoxicity evaluation in medulloblastoma patients treated with involved field boost using intensity-modulated radiation therapy (IMRT): a retrospective review

**DOI:** 10.1186/1748-717X-9-158

**Published:** 2014-07-21

**Authors:** Wilson Albieri Vieira, Eduardo Weltman, Michael Jenwei Chen, Nasjla Saba da Silva, Andrea Maria Cappellano, Liliane Desgualdo Pereira, Maria Ines Rabelo Gonçalves, Robson Ferrigno, Rodrigo Morais Hanriot, Wladimir Nadalin, Vicente Odone Filho, Antonio Sergio Petrilli

**Affiliations:** 1 Department of Radiation Oncology, Hospital Israelita Albert Einstein-HIAE, São Paulo, Brazil; 2 Departament of Radiation Oncology, Hospital das Clínicas da Faculdade de Medicina da Universidade de São Paulo-HCFMUSP, São Paulo, Brazil; 3 Department of pediatric oncology, Instituto de Oncologia Pediátrica/Grupo de Apoio ao Adolescente e à Criança com Câncer/Universidade Federal de São Paulo-IOP/GRAACC/UNIFESP, São Paulo, Brazil; 4 Department of speech therapy, Instituto de Oncologia Pediátrica/Grupo de Apoio ao Adolescente e à Criança com Câncer/Universidade Federal de São Paulo-IOP/GRAACC/UNIFESP, São Paulo, Brazil; 5 Department of pediatric oncology, Instituto de tratamento do cancer infantil (ITACI) do Hospital das Clínicas da Faculdade de Medicina da Universidade de São Paulo-HCFMUSP, São Paulo, Brazil

**Keywords:** Medulloblastoma, Hearing loss, Intensity-modulated radiotherapy, Cisplatin, Quality of life

## Abstract

**Background:**

Ototoxicity is a known side effect of combined radiation therapy and cisplatin chemotherapy for the treatment of medulloblastoma. The delivery of an involved field boost by intensity modulated radiation therapy (IMRT) may reduce the dose to the inner ear when compared with conventional radiotherapy. The dose of cisplatin may also affect the risk of ototoxicity. A retrospective study was performed to evaluate the impact of involved field boost using IMRT and cisplatin dose on the rate of ototoxicity.

**Methods:**

Data from 41 medulloblastoma patients treated with IMRT were collected. Overall and disease-free survival rates were calculated by Kaplan-Meier method Hearing function was graded according to toxicity criteria of Pediatric Oncology Group (POG). Doses to inner ear and total cisplatin dose were correlated with hearing function by univariate and multivariate data analysis.

**Results:**

After a mean follow-up of 44 months (range: 14 to 72 months), 37 patients remained alive, with two recurrences, both in spine with CSF involvement, resulting in a disease free-survival and overall survival of 85.2% and 90.2%, respectively.

Seven patients (17%) experienced POG Grade 3 or 4 toxicity. Cisplatin dose was a significant factor for hearing loss in univariate analysis (*p* < 0.03). In multivariate analysis, median dose to inner ear was significantly associated with hearing loss (*p* < 0.01). POG grade 3 and 4 toxicity were uncommon with median doses to the inner ear bellow 42 Gy (p < 0.05) and total cisplatin dose of less than 375 mg/m^2^ (*p* < 0.01).

**Conclusions:**

IMRT leads to a low rate of severe ototoxicity. Median radiation dose to auditory apparatus should be kept below 42 Gy. Cisplatin doses should not exceed 375 mg/m^2^.

## Introduction

Medulloblastoma is a common central nervous system (CNS) tumor in pediatric patients, accounting for 15-20% of all CNS tumors in this age group. Currently, the treatment for medulloblastoma consists of maximal resection, followed by postoperative radiotherapy (RT) of the intracranial and spinal subarachnoid volume, plus a boost to the posterior fossa (PF) or involved field (IF). Adjuvant cisplatin-based chemotherapy is also used. This approach results in a 5-year survival rate in up to 85% of standard risk (SR) cases [1-3].

Neurosensorial hearing loss (NSHL) is a common complication of treatment in children with medulloblastoma. Hearing loss impairs the academic and social development of these children [4]. Numerous studies have demonstrated that the severity of NSHL increases with higher RT doses to the inner ear [5,6]. Combined RT with cisplatin-based chemotherapy can enhance ototoxicity in children, mainly in high-frequency sounds. By minimizing the radiation dose to the inner ear, the risk of hearing loss can be reduced. Some studies have shown that the delivery of IF boost only instead of the whole PF achieve similar local control and survival rates compared to PF boost [7,8]. With the development of intensity-modulated radiation therapy (IMRT) it is now possible to further decrease the dose to normal tissues, including the inner ear in patients with medulloblastoma, thus potentially reducing the ototoxicity [9,10].

Hearing function is a complex human sense controlled by delicate structures that can be affected by radiation, whose impairment is attributed to changes in the cochlea or vasculature. It is hypothesized that NSHL results from cochlear damage [5-11]. The use of cisplatin in many patients also contributes to hearing loss, further complicating any attempt to determine a tolerance radiation dose.

Herein we performed a retrospective assessment of hearing function in a cohort of medulloblastoma children treated with IMRT. Our goal was to determine whether IF boost with IMRT can achieve a lower rate of ototoxicity and establish a threshold dose for the development of hearing loss. We also analyzed if the total cisplatin dose influenced the severity of NSHL.

## Methods

### Patients’ characteristics

This retrospective study was approved by the institutional review board. Patients were included in the study if they had: 1) normal hearing function at baseline; 2) treatment with IMRT for the boost volume; 3) follow-up ≥ one year; 4) age younger than 21. They were allocated to either standard risk (SR) or high risk (HR) groups. From February 2004 to August 2008, 41 patients with medulloblastoma were treated in the Department of Radiation Oncology of Hospital Israelita Albert Einstein (HIAE) and included in the study. These patients had maximal resection that could be safely performed, followed by adjuvant craniospinal irradiation (CSI) plus a boost to PF and/or IF, and adjuvant chemotherapy. IMRT was used to deliver the boost.

### Treatment by group stratification

All patients in the study were submitted to CSI with either conventional or conformal RT (3DRT) followed by a PF and/or IF boost with IMRT.

SR patients received 23 to 24 Gy CSI, followed by either a PF boost to 36 Gy and IF boost to 54 to 55.8 Gy (n = 10), or IF boost only to 55.8 Gy (n = 5). Five SR patients received 36 Gy CSI. HR patients were treated with 36 Gy CSI followed by an IF boost to 54 to 55.8 Gy.

For staging, CSI and IMRT boost planning and treatment, methods were similar as described by others [9,10], except for the planning system (Eclipse™/Varian INC, Palo Alto, CA).

For the study, dose-volume histograms were reviewed. Minimum, maximum, mean and median doses to the inner ear contoured in IMRT planning were obtained and correlated with hearing function.

Chemotherapy protocols for SR patients consisted of vincristine and etoposide during RT, followed by up to 8 cycles of cyclophosphamide, vincristine, and cisplatin six weeks later. Patients stratified as HR received 3 cycles of the same schema, before radiation followed by six months of oral etoposide. Patients with leptomeningeal spread received intravenous methotrexate. Twelve out of 20 SR patients received at least 6 cycles of cisplatin. Three patients, due to toxicity, received 4 cycles and 6 patients received carboplatin instead of cisplatin. In the HR group, 3 patients had carboplatin instead of cisplatin and only one patient had the last cycle cancelled due to hematologic complication. Mean cisplatin dose administrated on patients was 286.2 mg/m^2^ (range 0 to 600 mg/m^2^). Each patient’s record was analyzed individually to verify auditory apparatus delineation whose pattern consisted of a circular structure within the temporal bone including cochlea and semicircular channels [12].

### Hearing evaluation

All patients had normal hearing function at the beginning of RT. Pure-tone audiograms were used to assess hearing thresholds. The frequencies 250 Hz, 500 Hz, 1000 Hz, 2000 Hz, 3000 Hz, 4000 Hz, 6000 Hz and 8000 Hz were obtained and measured in decibel (dB) hearing level. Hearing function was graded on scale 0 to 4 according to Pediatric Oncology group’s (POG) [9] toxicity criteria. Patients with POG grade 3 and 4 toxicity were stratified as severe hearing loss group due to impairment in hearing speech frequencies and learning abilities. Meanwhile, those with normal hearing and POG grade 1 and 2 toxicity were considered in the non-severe group.

The last audiogram performed from the beginning of RT was considered for data analysis. Each ear was evaluated individually, however as no difference between sides was observed, a mean volume was calculated for data analysis.

### Outcomes

The primary outcome was to evaluate POG ototoxicity grade in medulloblastoma patients treated with IF boost using IMRT. Secondary outcomes were as follows: establish a relationship between the RT dose received by the inner ear with POG ototoxicity and the cumulative cisplatin dose with POG ototoxicity and analyze disease free and overall survival.

### Statistical analysis

POG’s ototoxicity grade was considered the final event for audiometric follow-up whereas recurrence and survival were considered end-points for disease free-survival and overall survival respectively.

Correlation between ear’s right and left variables were made by spearman correlation coefficient. A mean value was obtained from both ears for final data analysis.

Univariate analysis for comparison between severe and non-severe NSHL was performed with *t*-test for independent variables. For the multivariate analysis, a logistic regression model was used to study variables significance over severe NSHL likelihood. After adjustment of all variables, the least significant ones were excluded, resulting in reduced logistic regression model. ROC curves were used to discriminate variables efficiency in severe POG ototoxicity. Survival curves were estimated by Kaplan-Meier method.

## Results

Audiologic follow-up ranged from 12 to 71 months (mean of 41 months). Mean doses for minimum, maximum, mean and median in the inner ear were respectively: 37.85 Gy (range, 25.894 to 47.582 Gy), 48.325 Gy (range, 37.24 to 54.479 Gy), 43.665 (range, 28.085 to 50.973 Gy) and 43.605 Gy (range, 28.78 to 50.311 Gy) (Table 1). POG ototoxicity grade 0, 1, 2, 3 and 4 for the right and left ears were 29.3%, 46.3%, 7.3%, 12.2%, 4.9% and 28.2%, 43.6%, 10.3%, 12.8%, 5.1%, respectively. Thirty-four (82.9%) patients had POG grades 0 to 2 whereas 7 patients (17.1%) had severe ototoxicity (POG grade 3 or 4). Eleven (26.8%) patients had normal hearing function at the last audiogram (POG grade 0) (Table 2).

**Table 1:** Variables description

**Variable**	**N**	**Minimum**	**Maximum**	**Mean**
**Minimum dose (Gy)**	41	25.894	47.582	37.85
**Maximum dose (Gy)**	41	37.24	54.479	48.325
**Mean dose (Gy)**	41	28.085	50.973	43.665
**Median dose (Gy)**	41	28.78	50.311	43.605
**Audiologic follow-up (months)**	41	12.83	71.00	41
**Survival follow-up (months)**	41	14	72	44
**Age**	41	2.9	19.8	10

**Table 2:** Categorized hearing loss according to POG grade

**POG ototoxicity**	**Frequence**	**%**	**% accumulated**
0 - 2	34	82.9%	82.9%
3 - 4	7	17.1%	100%
Total	41	100%	

Mean cisplatin dose administrated on patients was 286.22 mg/m^2^; the drug was not given to 9 (22%) patients (Table 3).

**Table 3:** Mean cumulative cisplatin doses

**Cisplatin dose (mg/m**^**2**^**)**	**Frequence**	**%**	**% accumulated**
0	9	22%	22%
270	6	14.6%	36.6%
300	3	7.3%	43.9%
315	1	2.4%	46.3%
360	3	7.3%	53.7%
390	1	2.4%	56.1%
420	1	2.4%	58.5%
425	1	2.4%	61.0%
450	11	26.8%	87.8%
495	1	2.4%	90.2%
540	1	2.4%	92.7%
600	3	7.3%	100%
Total	41	100%	

Univariate analysis with *t*-test found no differences for the variables between the two groups studied, except for mean cumulative cisplatin dose (*p* < 0.01) (Table 4). Logistic regression model was performed in multivariate analysis in order to study all variables impact on severe ototoxicity (POG grade 3 and 4). Thereafter, the least significant variables were excluded in the reduced logistic regression model. Median RT dose to the auditory apparatus was a statistically significant factor for POG grade 3 and 4 (*p* = 0.012) whereas mean cumulative cisplatin dose may play an important role (*p* = 0.075) (Table 5).

**Table 4:** Univariate analysis for ototoxicity

	**POG ototoxicity**	**Frequence**	**Mean**	**Standard error**	***p*-value**
**Minimum dose**	0-2	34	3725.5	940.7	0.612
	3-4	7	3923.5	887.7	
**Maximum dose**	0-2	34	4819.9	400.2	0.851
	3-4	7	4849.8	246.0	
**Mean dose**	0-2	34	4315.3	480.1	0.296
	3-4	7	4513.8	241.6	
**Median dose**	0-2	34	4313.1	443.2	0.280
	3-4	7	4503.2	241.6	
**Mean cisplatin dose (mg/m**^**2**^**)**	0-2	34	288.68	196.759	0.003
	3-4	7	445.71	88.620	

**Table 5:** Reduced logistic regression model for POG ototoxicity grade 3 e 4

**Variable**	**Coefficient**	**Standard error**	**p-value**
**Median dose (Gy)**	−0.001	0.000	0.012
**Cisplatin dose (mg/m**^**2**^**)**	0.006	0.003	0.075

Cut-off points to determine variables efficiency were evaluated by ROC curves adjusted by reduced logistic regression model (Table 6). According to this analysis, cumulative cisplatin doses greater than 375 mg/m^2^ is an important risk factor for severe ototoxicity (*p* < 0.01) and median dose to auditory apparatus greater than 42 Gy increases patient’s chance to develop severe ototoxicity (*p* < 0.05).

**Table 6:** Reduced logistic regression model for cut-off points

**Variable**	**Coefficient**	**Standard error**	***p*-value**	**Odds ratio**
**Median < 42.04 Gy**	−2.191	1.180	0.043	0.112
**Cisplatin dose < 375 mg/m**^**2**^	−2.825	1.035	0.006	0.059

After a mean follow-up of 44 months (range, 14 to 72 months), 37 patients remained alive, with two recurrences, both in spine with CSF involvement, resulting in a disease free-survival and overall survival of 85.2% and 90.2%, respectively.

## Discussion

Our study shows that children with medulloblastoma can enjoy a lengthy good hearing function after treatment with postoperative chemoradiotherapy. The report of 17.1% of severe ototoxicity among the 41 patients compares favorably with the results of other studies [5-11,13] that have reported the outcomes of minimizing dose to the cochlea and hearing loss in medulloblastoma, besides being quite lower than the rate seen when conventional RT was used.

In our study, both cisplatin and RT doses were important risk factors in developing severe ototoxicity. The radiation limit dose for median auditory apparatus was 42 Gy and the cumulative cisplatin dose was 375 mg/m^2^, both in agreement with previous findings [9,10,13-15].

It is noteworthy that only 26.8% had no hearing deficit at all and were stratified as POG grade 0, a concern also noticed in other trials [5,6,8-11,13-15] that only few patients keep a normal hearing function in the long term, supporting the use of IMRT to reduce the severity of NSHL, allowing children to learn and develop normally.

In regard to survival rates, there was a concern among radiation oncologists that IMRT could compromise local control and survival because a greater conformality obtained with IMRT could jeopardize isodoses curves in the target. That was the reason why survival rates were analyzed in the present study, although the median follow-up was less than 60 months. Studies analyzing boost with 3DRT and IMRT and reduced volume obtained high survival and PF control rates [1,7,8,13], which is corroborated by our findings, highlighting the fact that IMRT is safe and doesn’t compromise local control and survival due to any geographical miss of the target.

The *t*-test results used in the univariate analysis were meant to be an introduction to a more sophisticated statistical multivariate model of logistic regression. There could be many reasons why the median dose to inner ear was not significant on univariate analysis: the small sample size of the group of patients who experienced the event (n = 7), the underlying distribution of the median dose to inner ear not being normal, among other reasons. The statistically significant result for the median dose to inner ear in the logistic regression analysis is stronger evidence that there is an association between this factor and the probability of a subject experiencing hearing loss than the non-significant result of a *t*-test comparing the median dose to inner ear of the two subgroups. Unfortunately, it was not possible to establish the onset of ototoxicity since not all patients did an audiogram on a regular schedule due to travel issues, a weakness in this study.

Researches from Texas Children’s Hospital reported 13% of ototoxicity in 15 medulloblastoma patients treated by boost IMRT compared with 64% in 11 patients treated by conventional RT [9]. This study had an update with a longer median follow-up of 41months and 44 patients evaluated and showed many similarities between our study [10]. POG grade 3 and 4 ototoxicity was seen in 25% of patients, whereas 25% of the patients had normal hearing function in both ears. All patients received cisplatin-based chemotherapy. Median onset for the development of POG grade 3 or 4 ototoxicity was 8.5 months after radiation, which is quite lower than what is expected when RT is the only treatment, that is, 2 to 4 years. There was a significant correlation in mean cochlear doses with severity of NSHL, aside from that; cochlear dose didn’t exceed 43 Gy in all ears with normal hearing function.

The biggest difference between both studies was that total cisplatin dose was not found in Paulino’s study to correlate with the degree of ototoxicity due to the fact that cisplatin dose was lowered when Grade 3 ototoxicity was encountered. Hence, patients who had less than Grade 3 ototoxicity had full doses of cisplatin and higher cumulative doses.

Nevertheless, cisplatin is the drug with the greatest ototoxicity potential known. Children are more prone to such hearing damage which depends on the dose, schedule and speed of infusion. On average, 50% of patients show some deficit in higher frequencies (6 and 8 Hz) with cumulative doses greater than 450 mg/m^2^. In children treated in combination with RT, this threshold dose is reduced substantially, with doses as low as 270 mg/m^2^ being associated with a high probability of NSHL. It has been observed that hearing acuity is either not affected or only minimally decreased in children treated only by RT [14,15]. As a matter of fact, doses to the inner ear less than 40 Gy hardly ever causes ototoxicity [11,14-16], however the threshold dose for NSHL with cisplatin-based chemotherapy and RT can be as low as 10 Gy [16-18]. Likewise, children with CNS shunting have increased risk to develop NSHL and the mean RT dose to the ear should to be limited in 45 Gy or even conservatively below 36 Gy, mainly when combined to cisplatin chemotherapy [11].

On the other hand, dose constraint below 35 Gy in the inner ear is only feasible in medulloblastoma patients with SR disease submitted to IF boost straight after 24 Gy CSI. Patients with HR disease, who need to be treated with 36 Gy CSI and those with SR disease whose boost is performed after a 36 Gy PF boost, usually receive doses above 40 Gy in the inner ear structures.

**Figure 1:**
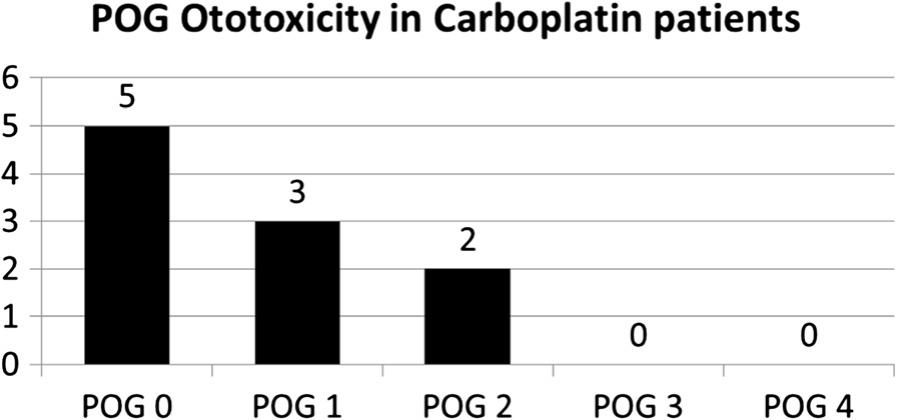
POG ototoxicity in patients who received carboplatin.

Our study was able to demonstrate that cisplatin plays a major role in the development of NSHL and is aggravated with increasing radiation dose to the cochlea. In the group of 7 patients with severe ototoxicity, mean cumulative dose was greater than in those whose hearing level was POG grade 0 to 2 (445.71 × 288.68 mg/m^2^). Moreover, none of the 9 patients who received carboplatin had severe hearing loss (Figure 1).

Therefore, we can infer from our findings and Paulino’s study that the benefit of dose reduction provided by IMRT is quite dependable on cisplatin cumulative dose. Considering the impact of cisplatin on survival, it is sine qua non to develop new strategies to decrease the side effects of chemoradiation in children. Hyperbaric oxygen treatment [19] and amifostin [20] has shown promising results in reducing the risk of post-treatment sequelae and will be our target for future trials.

## Conclusion

IMRT is a safe and valuable tool to reduce severe ototoxicity in medulloblastoma patients while achieving local control and survival rates comparable to conventional RT. RT and cisplatin doses should not exceed 42 Gy and 375 mg/m^2^, respectively.

## Competing interest

The authors declare that they have no competing interests.

## Authors’ contribution

The work presented here was carried out in collaboration between all authors. All authors read and approved the final manuscript.
